# Photochemotherapeutic strategies against *Acanthamoeba *keratitis

**DOI:** 10.1186/2191-0855-2-47

**Published:** 2012-09-05

**Authors:** Ruqaiyyah Siddiqui, Naveed Ahmed Khan

**Affiliations:** 1 Department of Biological and Biomedical Sciences, Aga Khan University, Karachi, Pakistan

**Keywords:** Acanthamoeba, Keratitis, Tin porphyrin, Treatment, Drug resistance, NMR

## Abstract

Here, we determined the potential of photochemotherapy, namely the application of photodynamic compounds followed by exposure to a suitable source of UV-visible radiation against corneal pathogen, *Acanthamoeba*. Organometallic macromolecule, tin porphyrin [Sn(IV)porphyrin] was synthesized and purity confirmed using nuclear magnetic resonance spectroscopy. The Sn(IV)porphyrin was tested against a keratitis isolate of *Acanthamoeba castellanii* belonging to the T4 genotype using growth and viability assays. The effects of Sn(IV)porphyrin on *A. castellanii* binding to and cytopathogenicity of human corneal epithelial cells *in vitro* were tested. The metalloporphyrin showed potent amoebistatic effects. The tin porphyrin inhibited amoebae binding to and cytopathogenicity of corneal epithelial cells. By using derivatives of photodynamic compounds [Sn(IV)porphyrin-antibody conjugates] for selective targeting of the parasite together with appropriate selection of light source will determine the potential of photochemotherapy against *Acanthamoeba* keratitis.

## Introduction

Due to increasing resistance of infectious micro-organisms to antimicrobial agents, it is becoming obvious to find alternative strategies for successful treatment of these devastating infections. Among others, *Acanthamoeba* keratitis is a serious human disease with sight-threatening consequences ([Khan, 2009]). Current treatment is problematic and involves hourly topical application of a cocktail of drugs including chlorhexidine, polyhexamethylene biguanide, propamidine isethionate, neomycin, and even then recurrence can occur ([Ficker et al., 1990]; [Perez-Santonja et al., 2003]). This is of particular concern in the absence of available alternative chemotherapeutic agents.

Photochemotherapy, namely the application of photodynamic compounds followed by exposure to a suitable source of UV-visible radiation, holds promise in future antimicrobial therapy ([Hamblin and Hasan, 2004]; [Huang et al., 2010]), particularly in eye infections due to ease of visible light usage. Here, we determined the potential of photodynamic compound, Sn(IV)porphyrin against a eukaryotic corneal pathogen, *Acanthamoeba*. Sn(IV)porphyrin is a synthetic metalloporphyrin that is a suitable tin porphyrin for catalytic and photocatalytic application because of the presence of a single metal oxidation state ([Sardana and Kappas, 1987]). Additionally, many derivatives can be formed with a variety of anions, which is important in the synthesis of an efficient photosensitizer. The formation of these derivatives is possible owing to the Sn(IV)centre, which is usually six-coordinate with two *trans*-diaxial ligands.

## Methods

### Synthesis of Sn(IV)porphyrin

Sn(IV)porphyrin was synthesized as follows: 250 mg of 5, 10, 15, 20-meso tetraphenyl porphyrine (TPP) and 200 mg stannous chloride (SnCl_2_.2H_2_O) were stirred and refluxed in 25 mL of pyridine in condensor at 115°C for 24 h. Approximately, 150 mL H_2_O was added and the solution was allowed to cool at room temperature. The crystals of Sn(IV)porphyrin were collected by vacuum filtration, washed with water and dried by vacuum suction yielding 250 mg of product, which showed maximum solubility in DMSO. The stock solution of Sn(IV)porphyrin was maintained in 10 mM concentration and stored in −4°C.

### Growth and viability assays

The effects of Sn(IV)porphyrin on *Acanthamoeba* viability and growth were determined. A clinical isolate of *A. castellanii* from a keratitis patient (ATCC 50492) was grown in PYG and incubated with Sn(IV)porphyrin (10, 50, 100 μM) in 24-well plates (2.5 x 10^5^ amoebae/mL/well) for 24 h under visible light. Amoebicidal and amoebistatic effects were determined using haemocytometer counting ([Siddiqui et al., 2012]) and Trypan blue exclusion testing ([Thorson et al., 1995]). For controls, amoebae were incubated with solvent alone (i.e., DMSO). Normal growth rates were determined using growth medium alone and considered as 100%. The results were presented as relative percent of *A. castellanii* incubated in PYG. Experiments were performed in duplicate and repeated at least three times.

### Adhesion assays

Adhesion assays were performed to determine the effects of Sn(IV)porphyrin on *A. castellanii* binding to human corneal epithelial cells (HCEC). HCEC were grown to confluency in 24-well plates in RPMI-1640 containing 10% foetal calf serum and 2 mM glutamine ([Sissons et al., 2005]; [Araki-Sasaki et al., 2000]). *A. castellanii* (10^6^ amoebae/well) were pre-incubated with various concentrations of Sn(IV)porphyrin or solvent alone for 45 min under visible light and then added to cell monolayers for 1 h and percentage binding determined as follows: 100 – [no. of unbound amoebae/total number of amoebae x 100] =% bound amoebae.

### Cytopathogenicity assays

Cytopathogenicity assays were performed to determine effects of Sn(IV)porphyrin on *A. castellanii-*mediated HCEC death. Amoebae were pre-incubated with Sn(IV)porphyrin or solvent alone as per adhesion assays. Amoebae were collected by centrifugation and resuspended in RPMI 1640 without Sn(IV)porphyrin and added to cell monolayers. Plates were incubated at 37°C in a 5% CO_2_ incubator for 24 h. Cytopathogenicity was determined by measuring lactate dehydrogenase release as follows: (sample value – control value/total LDH release – control value x 100 =% cytopathogenicity) ([Sissons et al., 2005]). Control values were obtained from HCEC incubated in RPMI alone. Total LDH release was determined from HCEC treated with 5% Triton X-100 for 30 min at 37°C.

## Results

### NMR spectroscopy confirmed the purity of Sn(IV)porphyrin

To confirm purity of Sn(IV)porphyrin, octahedral Sn(IV) complex, TPPSnCl_2_ was dissolved in C6D6 solvent and studied for chemical shifts and couplings constants between different position of hydrogen atoms. The calculated *meso* proton chemical shift for TPP complex was 9.02 ppm and average coupling constant *J*(SnH) was found to be 14.9 Hz. The results calculated from NMR spectrum were tallied with experimental study of where their chemical shift was 9.18 (our experiment revealed 9.02) and average coupling constant was 15.2 Hz (our experiment revealed 14.7 Hz), indicating similarities in the structure of the molecule and the number of neighboring NMR active nuclei within the molecule. Overall, NMR spectrum revealed chemical shift (σ) at *meso* proton signal (β pyrrole) to be 9.02 ppm. Average of ^119^ Sn-^1^H and ^117^Sn-^1^H coupling constants, i.e., *J* (SnH) to *meso* proton (β pyrrole) was 14.7 Hz.

### Sn(IV)porphyrin inhibited *A. castellanii* growth but no effect on its viability

At micromolar concentrations, Sn(IV)porphyrin exhibited significant amoebistatic effects compared with amoebae incubated with solvent alone (*P <* 0.05, as standard level of significance, when Sn(IV)porphyrin-treated samples were compared with solvent-treated samples using *T*-test, paired, one-tail distribution) (Table 1). Both 10 μM and 50 μM concentrations of Sn(IV)porphyrin inhibited amoebae growth by 51% ± 6.3 and 73% ± 4.4 respectively, while solvent alone inhibited *A castellanii* growth by 12% ± 2.1(Table 1). In contrast, Sn(IV)porphyrin had no effect on the viability of *A. castellanii* (Table 1).

**Table 1:** **Effect of Sn(IV)porphyrin on biological properties of *Acanthamoeba castellanii***

	**% Growth inhibition**	**% Viability inhibition**	**% Adhesion inhibition**	**% Cytopathogenicity inhibition**
*Acanthamoeba *+ solvent	12%±2.1	2.1%±1.1	6.8%±0.4	11.7%±0.8
*Acanthamoeba *+ Sn(IV)porphyrin (10μM)	51%±6.3	4.5%±0.3	12.7%±2.3	15.4%±2.2
*Acanthamoeba *+ Sn(IV)porphyrin (50μM)	73%±4.4	7.3%±2.2	44%±4.1	58.8%±6.2
*Acanthamoeba *+ Sn(IV)porphyrin (100μM)	98.2%±5.3	12.7%±1.3	67%±3.2	72.3%±5.2

Data are presented as the mean ± standard error of three independent experiments performed in duplicate.

### Sn(IV)porphyrin inhibited *A castellanii* adhesion to and cytopathogenicity of human corneal epithelial cells

The results revealed that Sn(IV)porphyrin significantly reduced amoebae binding to HCEC monolayers, compared with amoebae incubated with solvent (*P <* 0.05 as standard level of significance using *T*-test, paired, one-tail distribution) (Table 1). At 50 μM and 100 μM concentrations, Sn(IV)porphyrin inhibited amoebae binding to HCEC monolayers by 44% ± 4.1 and 67% ± 3.2 respectively. *A. castellanii* incubated with solvent had no effect on adhesion of amoebae to HCEC. Cytopathogenicity assays were performed to determine the effect of Sn(IV)porphyrin on *A. castellanii*-mediated HCEC death. *A. castellanii* alone produced 85% ± 3.8 HCEC death within 24 h. With solvent, *A. castellanii*–mediated HCEC death was inhibited by 11.7% ± 0.8. In the presence of 50 μM Sn(IV)porphyrin, *A. castellanii*-mediated HCEC cytopathogenicity was significantly inhibited 58.8% ± 6.2 (*P <* 0.05 when Sn(IV)porphyrin-treated samples were compared with solvent-treated samples using *T*-test, paired, one tail distribution), which was further reduced to 72.3% ± 5.2 at 100 μM of Sn(IV)porphyrin respectively.

## Discussion

For the first time, these studies indicate that organometallic macromolecule, tin porphyrin [Sn(IV)porphyrin] exhibit potent amoebistatic effects suggesting that photochemotherapy holds promise in future antimicrobial therapy, particularly in eye infections due to ease of visible light usage. The use of visible light in our findings is particularly significant as the present mode of eye inspection is visible light. The basis of this technology is that visible light (or light of appropriate wavelength) activates photosensitising compound, resulting in production of singlet oxygen and other reactive oxygen species (ROS) to induce cell death in the target pathogen/tissue. If photosensitising compounds can be specifically delivered to the pathogen, there will be several advantages over the present treatment of *Acanthamoeba* keratitis, which is laborious and involves hourly topical application of mixture of drugs including chlorhexidine, polyhexamethylene biguanide (PHMB), neomycin and propamidine isethionate that can last up to a year with associated side effects and even then recurrence can occur ([Khan, 2009]). In the present study, the synthetic Sn(IV)porphyrin macromolecule incorporated a closed shell Sn(IV) into the porphyrin molecule affecting the ground state optical spectrum and fluorescence spectrum. This photophysical or photosensitizing property of the diamagnetic complex i.e., Sn(IV)porphyrin shows an increased triplet state lifetime important for photoactive damage ([Land et al., 1988]).

Targeted treatment of eukaryotic pathogen remains a major problem in our efforts to counter infectious diseases. As opposed to bacterial pathogens with unique targets, the molecular and functional similarities in eukaryotic pathogens to human cells make it particularly challenging to find novel targets for therapeutic interventions. The synthesis of derivatives of these photodynamic compounds in order to improve their selective attachment to the parasite is a complete new approach to this infection. To this end, studies have shown the presence of an adhesin, mannose-binding protein (MBP) on the surface membranes of *Acanthamoeba* that is critical in its pathogenesis ([Alsam et al., 2003]; [Garate et al., 2004]). For robust and targeted killing, future studies will synthesize photosensitizer-alpha-D-mannose conjugates of varying charges as well as photosensitizer-MBP antibody conjugates. This together with appropriate selection of light source (lasers) to emit monochromatic light in the visible spectrum in a given direction targeting and fully activating the photosensitizer compound will determine the potential of photochemotherapy to counter this devastating infection. In addition, these studies will provide a model and stimulate ongoing research in targeting other pathogens, which cause serious eye infections.

### Ethical approval

Not required.

## Competing interest

The authors declare (1) no conflicts of interests for the submitted work; (2) no financial relationships with commercial entities that might have an interest in the submitted work; (3) no spouses, partners, or children with relationships with commercial entities that might have an interest in the submitted work; and (4) no non-financial interests that may be relevant to the submitted work.

## Funding

This work was supported by Life Science grants from Birkbeck, University of London, the Higher Education Commission, Pakistan and the Aga Khan University.
